# The War Office and Its Temporary Hospitals

**Published:** 1915-04-17

**Authors:** 


### THE WAR OFFICE AND ITS TEMPORARY HOSPITALS.

Many asylums and Poor-Law infirmaries, both in London and the provinces, have now been taken over by the War Office for the treatment of wounded soldiers. Unfortunately, no uniform system of administration has been adopted. The demands upon the War Office are so heavy that it has been found impossible to run them upon the same lines as the permanent military hospitals. It has, therefore, been found necessary to make arrangements which vary greatly in detail in different localities. The ruling principle adopted has been to introduce the military system of internal administration as regards discipline, the discharge of patients, and the keeping of accounts, and to enlist the services of the local governing body for the supply of provisions, drugs, and necessaries. The main differences which have arisen are found in the extent of the powers given to the officer in charge and of those retained by the governing body. The best system is that which has been selected in the case of the Cardiff Mental Hospital, and was described in our columns two weeks ago. Here the hospital and the whole of the staff have been taken over by the War Office, which will appoint any additional staff required. The medical superintendent will be the officer in charge under the direct control of the War Office. He will make contracts for supply, and otherwise carry on the business side of the administration, and will present monthly accounts to the War Office through the Board of Control. In Bethnal Green the Guardians have decided to take no part in the administration, and have retained the whole of their staff with the exception of the matron and a few of the nurses and domestic servants. The War Office will, therefore, administer the new hospital directly, and its medical staff will be derived, to a large extent, from the London Hospital. Mr. Hurry Fenwick will be the officer in charge. At the other London infirmaries which have been taken over the medical superintendent will be given a commission, and will be made the officer in charge. The Guardians will continue to make contracts, and will supply drugs, provisions, and necessaries, and will undertake any structural alterations required. The additional staff, with the exception of the medical officers, will also be obtained through the Guardians. This is, we think, a mistake. It would be better that the officer in charge should be made responsible for the selection of the staff, subject only to the control of the War Office.

The salaries and emoluments of the officers taken over from the Guardians are to remain unaffected by the change, and provision has been made to safeguard the interests of the officers whose services will not be required. The position as regards the nursing staffs is far from satisfactory. In the case of the asylums, sisters and charge nurses are now being advertised for at Army rates; in some of the infirmaries an attempt is being made to obtain nurses on the Poor-Law scale, which is considerably lower. This will certainly lead to much dissatisfaction and to difficulty in obtaining suitable candidates. Under the Army system of administration the sister holds a more responsible position than is the case in a Poor-Law infirmary, and she should therefore receive a higher salary.

Care has been taken by the War Office to remove from the infirmaries taken over all notices or other indications of their former connection with the Poor Law. The significance of this has not been realised by all Boards of Guardians. One London Board has issued a statement that the military hospital will continue under the control and management of the Board of Guardians and has appointed a hospital committee for the purpose of its administration. This is certainly not in accordance with public sentiment. Great as has been the improvement in the Poor-Law administration of infirmaries in recent years, its defects are still very great. A system of dual military and civil control is bound to lead to friction and unnecessary delay in the execution of needed improvements. The general public opinion will not tolerate the association of the wounded with the evil reputation which still clings to the Poor-Law Guardians. It may be necessary, in order to save the time which a complete severance would involve, that the Guardians should be allowed to continue to make contracts and act as paymasters for the War Office, but the administration of the hospitals should be removed entirely from their hands.

